# Leadership and training

**DOI:** 10.1007/s10353-016-0421-5

**Published:** 2016-05-12

**Authors:** D. Öfner

**Affiliations:** Department of Visceral-, Transplant and Thoracic Surgery, Center of Operative Medicine, Innsbruck Medical University, Anichstraße 35, Innsbruck, 6020 Austria

**Keywords:** Surgical training, Working hours, Work-life-balance

## Abstract

Against the background of substantial changes in the field of healthcare in Austria, the specialization in surgery must be reconsidered starting from modified points of view. However, in this context, the new training regulations are not the only standard: the training officers must show leadership skills by paying attention to the modified framework conditions and by promoting a new corporate culture related to training with innovating concepts. The challenge of the threatening quality loss in surgery can only be addressed in this way.

## Changing framework conditions

The Austrian healthcare system faces a significant radical change. Many speak about a crisis concerning not only the training of young physicians under the Austrian Working Time Act with the restriction of working hours, but also almost all fields of medicine and, particularly heavily, the field of surgery [1]. This is affected particularly heavily because training in the field of surgery and the profession itself offer very little in terms of *“work–life balance”* and training until now has been poorly organized structurally. Training in surgery and the profession of surgeon are physically and mentally demanding and therefore less attractive. It is no secret that practice makes perfect. In a field like surgery, where manual dexterity also is required, training should leave sufficient time for practice. And, to assure quality, this time must also be available in doctors’ future professional life. The connection between quality and quantity in surgery is not only proven for individual surgery departments (hospital volume), but also for the surgeons themselves (surgeon volume) on hundreds of thousands of patients. The fact that, in addition to this, the workplace is also less lucrative, because of the different regional and international wage levels, is another factor contributing to the lack in general of junior staff surgeons, attracting young surgeons almost only to the health institutions of larger cities. This will lead to further centralization which, although desirable in many fields, brings the risk that the primary surgical care of the population—in analogy to the primary nursing sector with general practitioners in the countryside—cannot be born only by central hospitals. Austria has one of the highest densities of physicians and beds in the world, and this situation will inevitably change in future. The drivers of this development, which shake the core of a rigid health system, are generally known. Firstly, they relate to the exponentially growing medical-technical progress, the increasing digitalization of medicine with integration of medical products in existing data and communication networks, the political and social priority change, the increasing financial pressure and the demand for quality assurance. Secondly, they concern demographics, the expected disproportionate increase of dementia patients, the de-solidarization of insurance systems and growing patient autonomy. Already, there are visible changes in personnel structure: in the medical field more women are employed. This forcedly leads to changes in the working conditions, as adequately true-to-life working models must be offered. The changed political and social priorities include the fact that in all post-industrial societies, the demand for healthcare services is increasing and the priority for investments are more focused on education and less on health. The pressure to offer good medicine at acceptable prices is ever-present, and the public sector as a financial contributor is neither able nor wants to constantly counter-finance the increasing costs and outflows. In the insurance branch, the co-payment percentage grows, and the tariff structures are modified by risk selection. Thus, multiclass medical care has been a reality for a long time now, and the second healthcare market is growing disproportionately. Between these cornerstones of changes, employees in the meantime choose their jobs by precisely demanding further training, job quality, motivation, recognition, remuneration, de-bureaucratisation and work–life balance. However, they are thus confronted with a fundamental change in medical working tasks (promoted by KA-AZG, the Austrian working hours act), a training period prolonged by legal requirements, still over-boarding administration work, increasing pressure in the direction of early specialization, increasing mechanization of the working world, which in part relieves, but at the same time alienates the work and creates pressure to always work more and faster (the rat race) and, if possible, without making any mistakes.

## A way out of the crisis by leadership

Although one of the wisdoms often used in management seminars, namely that a crisis is also a chance to improve, is not exactly right, it does have its validity. And you do not need to wander to the Far East to recognize the double face of the crisis: even the Greek term *“krisis”* does not designate a hopeless situation, but the climax and turning point of a dangerous situation, starting from which you cannot do anything other than improve. Against the background of these challenges in the medical field, in this era of radical change there is a high demand for *leadership*. In the medical field, *leadership* has many semantic facets; from the assumption of responsibility, to the advocacy of the patient, to the avoidance of defensive medicine, teamwork, transparency, communication, positive error culture and so forth. Regarding training, it must be noted that the responsibility primarily lies on-site, and, as a consequence, with the primary physicians, and it can just be supported by the hospital owner, the societies and the Medical Chamber. Therefore, there is a demand for leadership in training, first of all of primary physicians. It is their task to responsibly care about training. In this context, a structured, transparent training plays a central role. The new training regulations help in this context. On one hand, because of the restrictive working time set by KA-AZG, it is pleasing that in the surgical training after basic training, the complete further training time can be passed at the surgery working station. On the other, practical knowledge with regard to other medical disciplines, which play a pivotal role in the era of multimodal therapy, is mandatory. Those, for example, interested in surgical oncology, cannot renounce pathology, radio-oncology, and internal oncology during training. In this field, there is a high demand for leadership for primary physicians who should profit from the possibility to develop and offer a respective curriculum including rotation opportunities. A further core task concerning leadership consists of creating framework conditions, spaces and chances for self-organization in training, so that employees can organise themselves alone to the greatest possible extent. Clinics with engaged employees create more output, and it is well-documented scientifically that there is a correlation between engagement and thinking of employees and the highest ranking of enterprises. Clinics with these attributes will also not have any problem with the recruitment of young physicians, even against the background of the modified signs described above. Supervisors must responsibly handle the time available for training. According to surveys, young surgeons often express that they are not allowed to attend further education events because of the argument that they are indispensable for routine operations or because senior physicians are privileged. This is a circumstance that seems completely out of the question in the context of an inflationary number of further education events. In the end, a corporate culture shaped by leadership with a differentiated attitude to error tolerance is also a question of the human image.

The opportunities on site, offered by the training officer, are not exhaustive but should be supplemented by the employer. The employer is responsible for the creation of workplace conditions reflecting appropriate, true-to-life working models, like the organization of a crèche in the hospital area or a suitable range of very differentiated working time models. The first point consists of diffusing many opportunities and difficulties connected with a return to working life after maternity leave, even during the training. Two thirds of all young physicians complain about over-boarding administrative tasks that are not directly related to their training. It is a simple task of the management of a clinic to delegate this job to nonmedical personnel. The further shifting of adequate tasks to other professional groups, like the nursing sector, will not be counterproductive for the quality of training of young physicians. Especially in the nursing sector, adequate potential for this reorganization can be found in the sense of “co-responsible area of activity” and, in addition, as extended competences in the nursing sector, which must of course be connected to corresponding training of this professional group, including the academization of the nursing sector. In other countries, such as the UK, this model has been successfully implemented for a long time, to the satisfaction of all parties.

In conclusion, in the recent past the further education academy of the Austrian Society for Surgery (Österreichische Gesellschaft für Chirurgie) has set accents in this sense. By using various different media, a transparent, open communication and information platform was established. After an initial hesitant start, the platform is now increasingly used. It should put together all the surgeons training in Austria. In this way, a platform transmitting the most differentiated information in the form of digital tools, such as blogs etc., was created. In addition, this platform provides learning materials and puts information on training issues etc. at users’ disposal. The rich offer of further education seminars, which are reported separately by the further education academy are also held during suitable congresses and conventions, will remain unchanged. In this way, the training is also structured by the further education academy.

## Conclusion

Thus, in the field of training there is a demand for leadership in order to reform the training according to Humboldt’s educational ideals, making it possible to efficiently address the threat of quality loss in surgery.
